# Nestin predicts a favorable prognosis in early ampullary adenocarcinoma and functions as a promoter of metastasis in advanced cancer

**DOI:** 10.3892/or.2014.3588

**Published:** 2014-11-04

**Authors:** YAN-SHEN SHAN, YI-LING CHEN, MING-DERG LAI, HUI-PING HSU

**Affiliations:** 1 Department of Surgery, National Cheng Kung University Hospital, Tainan, Taiwan, R.O.C.; 2 Institute of Clinical Medicine, College of Medicine, National Cheng Kung University, Tainan, Taiwan, R.O.C.; 3 Department of Senior Citizen Service Management, Chia-Nan University of Pharmacy and Science, Tainan, Taiwan, R.O.C.; 4 Department of Biochemistry and Molecular Biology, College of Medicine, National Cheng Kung University, Tainan, Taiwan, R.O.C.; 5 Institute of Basic Medical Sciences, College of Medicine, National Cheng Kung University, Tainan, Taiwan, R.O.C.; 6 Center for Infectious Diseases and Signaling Research, College of Medicine, National Cheng Kung University, Tainan, Taiwan, R.O.C.

**Keywords:** ampullary adenocarcinoma, nestin, cyclin-dependent kinase 5, distant metastasis, Ingenuity Pathway Analysis

## Abstract

Nestin exhibits stemness characteristics and is over-expressed in several types of cancers. Downstream signaling of nestin [cyclin-dependent kinase 5 (CDK5) and Ras-related C3 botulinum toxin substrate 1 (Rac1)] functions in cancer to modulate cellular behaviors. We studied the function of nestin in ampullary adenocarcinoma. Immunohistochemistry (IHC), reverse transcription-polymerase chain reaction, and cDNA microarray of nestin in ampullary adenocarcinoma was compared with normal duodenum. CDK5 and Rac1 were assessed by western blotting. We hypothesized that nestin/CDK5/Rac1 signaling behaves different in early and advanced cancer. We found that the presence of nestin mRNA was increased in the early stages of cancer (T2N0 or T3N0) and advanced cancer with lymph node metastasis (T4N1). A total of 102 patients were enrolled in the IHC staining. Weak nestin expression was correlated with favorable characteristics of cancer, decreased incidence of local recurrence and lower risk of recurrence within 12 months after surgery. Patients with weak nestin expression had the most favorable recurrence-free survival rates. Patients with mild to strong nestin expression exhibited an advanced behavior of cancer and increased possibility of cancer recurrence. The reciprocal expression of nestin and RAC1 were explored using a cDNA microarray analysis in the early stages of ampullary adenocarcinoma. Increased level of CDK5 with simultaneously decreased expression of Rac1 was detected by western blotting of ampullary adenocarcinoma in patients without cancer recurrence. The activation of multiple oncogenic pathways, combined with the stemness characteristics of nestin, formed a complex network in advanced ampullary adenocarcinoma. Our study demonstrated that nestin performs a dual role in ampullary adenocarcinoma. Appropriate amount of nestin enhances CDK5 function to suppress Rac1 and excessive nestin/CDK5 participates in multiple oncogenic pathways to promote cancer invasiveness. Inhibiting nestin in patients who exhibit nestin-overexpressed ampullary adenocarcinoma may be a method of preventing cancer recurrence.

## Introduction

According to the Surveillance, Epidemiology, and End Results program in the United States, the 10-year survival rate of patients with ampullary cancer after resection is 41.8% (1). Combining surgery and chemoradiotherapy can improve survival rates; however, the 3-year survival rate among patients with metastatic cancer is only 20% (2). Several histopathological markers have been identified that predict prognosis, including tumor stage, nodal metastasis, lymphovascular invasion, pancreaticobiliary subtype, pancreatic invasion and resection margin (3–5). Despite these predictors, the metastasis of ampullary cancer is unpreventable.

The molecular mechanisms of carcinogenesis in ampullary cancer include the inhibition of tumor suppressors, mutation of oncogenes, depletion of adhesion molecules, infiltration of tumor-associated macrophages and activation of cancer stem cells (6–10). Cancer stem cells (CSCs) remain dormant to escape from chemoradiotherapy (11). The cytoskeleton is modified in metastatic CSCs; this process has been termed epithelial-mesenchymal transition (EMT) (12,13). Scholars have observed the concomitant expression of EMT- and CSC-related genes (14–16). Certain stemness factors are modified during EMT of cancer (13,14). Nestin is an intermediate filament protein that exhibits stemness characteristics in brain tumors. Nestin^+^/CD133^+^ tumor cells are regarded as CSCs (17). Nestin has been found to be overexpressed in several types of cancers (18–20). Therefore, nestin provides a link between CSCs and EMT, and overexpression of nestin may play a role in cancer metastasis. This study assessed the functions of nestin in ampullary adenocarcinoma and its correlation with clinical outcomes of patients. We hypothesize that nestin modulates cancer progression and is associated with patient survival.

## Materials and methods

### Patients

A total of 102 patients who were diagnosed with ampullary adenocarcinoma and who underwent radical resection from January 1990 to April 2013 at the National Cheng Kung University Hospital (NCKUH) were enrolled. Patients who received conservative treatment or exhibited other cell types of ampullary cancer were excluded. Demographics, histopathological information and clinical outcomes were collected by conducting a retrospective review of the patient charts.

### Immunohistochemical (IHC) staining

Tissue of ampullary adenocarcinoma and the normal duodenum were fixed in 4% formalin and embedded in paraffin. Serial sections were cut from each sample. All samples were acquired from the Human Biobank at the Research Center of Clinical Medicine of NCKUH after obtaining appropriate informed consent. The study was approved by the Institutional Review Board (NCKUH IRB no: A-ER-100-395).

IHC staining was performed using a monoclonal mouse anti-human nestin antibody (Santa Cruz Biotechnology, Inc., Dallas, TX, USA). The sections were incubated using an avidin-biotin complex reagent (Dako, Carpinteria, CA, USA) and final color was developed with 3-amino-9-ethyl carbazole (Zymed Laboratories, Inc., San Francisco, CA, USA). The sections were counterstained with hematoxylin. The internal positive controls consisted of endothelial cells, and primary or secondary antibodies were omitted to serve as negative controls. The immunoreactivity of the nestin protein was assessed using the semi-quantitative method and scaled according to the immunoreactive score (IRS) established by Remmele and Schicketanz (21). The IRS points ranged from 0 to 12 and were divided into 4 classifications: negative, weak, mild and strong. One researcher assessed all lesions (H.-P.H).

### Semi-quantitative reverse transcription polymerase chain reaction (RT-PCR)

The fresh cancer tissues and normal duodenum were obtained for RT-PCR. The total RNA was extracted from the fresh tissues, and single-stranded complementary DNA (cDNA) was synthesized using oligo(dT) as the random primer. The cDNA was amplified using the primers for β-actin and nestin genes: β-actin sense, 5′-AGC GGG AAA TCG TGC GTG-3′ and antisense, 5′-CAG GGT ACA TGG TGG TGG TGC C-3′; nestin sense, 5′-TTG ACC AGG AGA TAG CTA GAC CTC-3′ and antisense, 5′-GAC TTT CCT TGT CTA CCT CCT CTG-3′. The RT-PCR products were analyzed using agarose gel electrophoresis, and the nestin bands were semi-quantified using densitometric analysis and subsequently normalized relative to the β-actin bands.

### cDNA microarray

Fresh tissues of paired ampullary adenocarcinoma and normal duodenum were analyzed using a cDNA microarray study. The RNA from the normal duodenum was labeled with Cy3 (PerkinElmer, Waltham, MA, USA) and the RNA from the ampullary adenocarcinoma was labeled with Cy5 during the *in vitro* transcription process. The Cy-labeled complementary RNA was hybridized to an Agilent SurePrint G3 Human GE 8×60K microarray (Agilent Technologies, Santa Clara, CA, USA). The microarrays were scanned at 535 nm for Cy3 and 625 nm for Cy5. The scanned images were assayed and substantially normalized using the rank-consistency filtering Lowess method. The data were analyzed using GeneSpring software. An Ingenuity Pathway Analysis (IPA 6.0; Ingenuity Systems, Mountain View, CA, USA) was used for the networks of the interacting genes.

### Western blotting

Total protein lysates from the tumor specimens or cells were obtained, and the protein concentration of the supernatants was measured using the amido-black method. Equal amounts of protein (30 μg) were separated on 10–15% polyacrylamide gels by SDS-gel electrophoresis and transferred to polyvinylidene difluoride membranes. Immunodetection was performed using an antibody against interacting proteins of nestin [cyclin-dependent kinase 5 (CDK5); Ras-related C3 botulinum toxin substrate 1 (Rac1)] and α-tubulin (Cell Signaling Technology, Inc., Danvers, MA, USA). Protein expression was visualized by ECL chemiluminescence (Promega, Madison, WI, USA) and quantitated by comparison with α-tubulin.

### Statistical analysis

All statistical analyses were conducted using SPSS version 12.0 (SPSS Inc., Chicago, IL, USA). A univariate analysis of the categorical variables was performed using the Chi-square test. The continuous variables were compared using the nonparametric Kruskal-Wallis H test. Any association between specific markers and the recurrence-free survival of patients was assessed using the Kaplan-Meier method, and the level of significance was tested using the log-rank test. The Cox proportional hazard regression model was used to evaluate multiple predictors of recurrence-free survival. Each model included age and gender as covariates. A P-value <0.05 was considered statistically significant.

## Results

### Expression of nestin mRNA in ampullary adenocarcinoma

**Figure 1:**
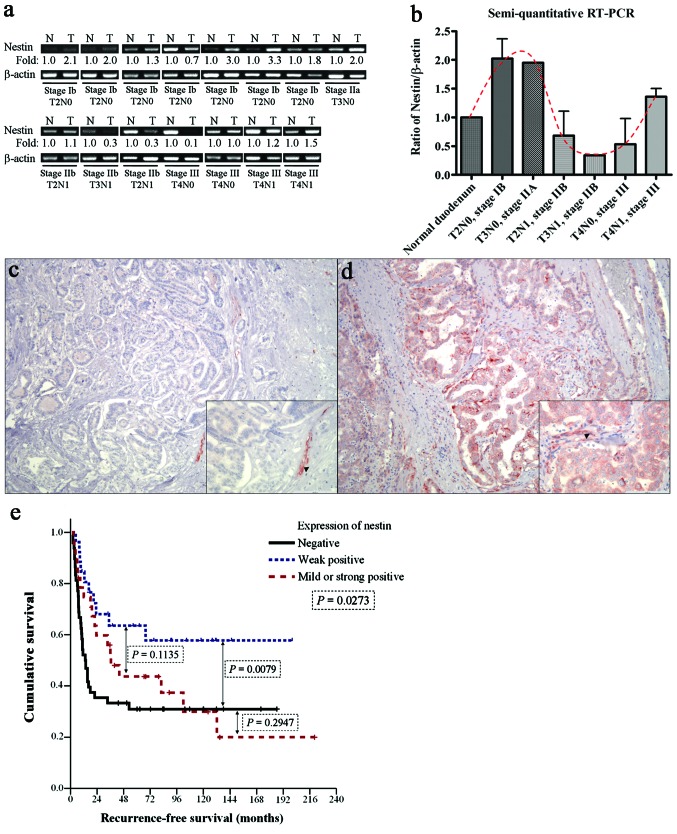
Expression of nestin in ampullary adenocarcinoma by semi-quantitative RT-PCR and immunohistochemistry (IHC) staining. (a) Fresh tissue samples from ampullary adenocarcinoma (T) and corresponding normal duodenum (N) were collected from different stages of disease. A total of 15 pairs of samples were assessed. β-actin served as a loading control. The fold-change of nestin/β-actin is labeled below the band. (b) The cancer/normal proportion of nestin/β-actin expression is correlated with pathological stage. (c and d) Expression of nestin in ampullary adenocarcinoma by IHC staining. The main images were captured at ×100 magnification, and the small images located at the right lower quadrant of each figure were captured at ×400. (c) Negative expression of nestin in ampullary adenocarcinoma (T2N1, stage IIB). The arrowhead indicates new lymphatic ducts or vessels within the tumor. (d) Dense expression of nestin in ampullary adenocarcinoma (T3N0, stage IIA) and a lymphovascular duct, indicated by an arrowhead in the magnified view. (e) Kaplan-Meier recurrence-free survival analysis of nestin expression in patients with ampullary adenocarcinoma (log-rank test) (P=0.0041). Solid black line represents the survival curve of patients with negative expression of nestin, fine dotted blue line represents the survival curve of patients with weak positive expression of nestin and the coarse dotted red line represents the survival curve of patients with mild to strong positive expression of nestin

The level of nestin mRNA expression was detected in the 15 pairs of cancer and normal tissue samples (Fig. 1a). Fig. 1b shows the quantitative ratio of nestin to β-actin expression. An increased nestin:β-actin ratio was found in the early stages of cancer without lymph node metastasis (T2N0 or T3N0) and in advanced cancer with lymph node metastasis (T4N1). However, the nestin:β-actin ratio was low in T2N1, T3N1 and T4N0 cancers (Fig. 1a). The fluctuating curve shown in Fig. 1b demonstrated the various function of nestin in early and advanced ampullary adenocarcinoma.

### Expression of nestin protein in ampullary adenocarcinoma

**Figure 2:**
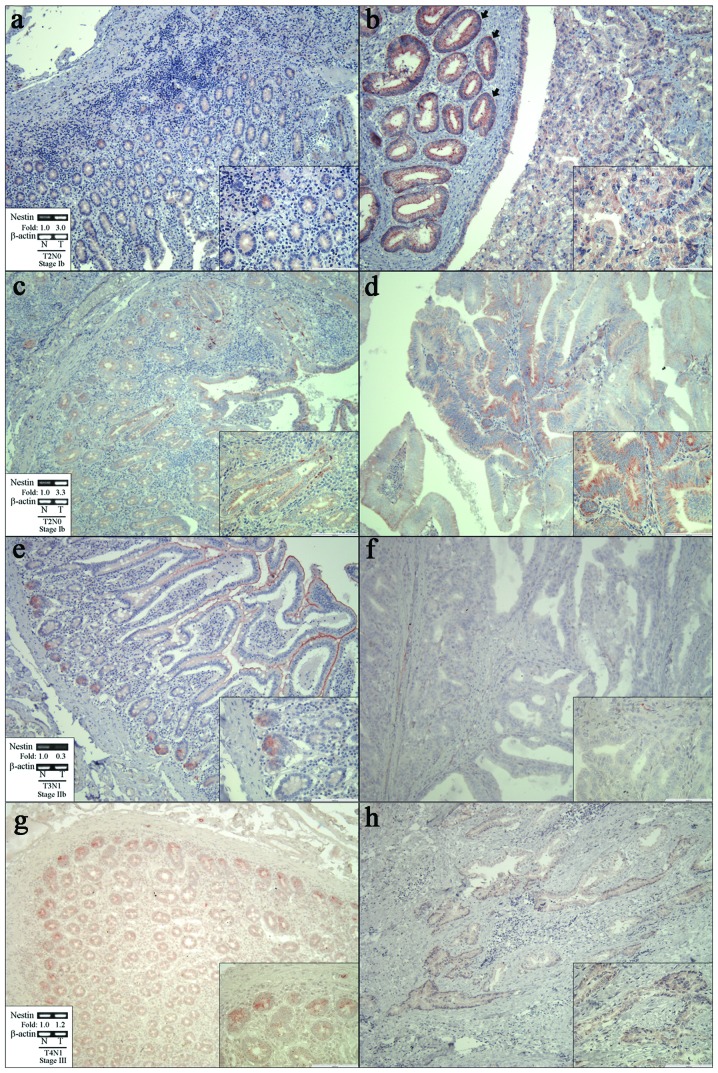
Expression of nestin in ampullary adenocarcinoma by immunohistochemistry (IHC) staining. The main images were captured at ×100 magnification and the small images located at the right lower quadrant of each figure were captured at ×400. (a, c, e and g) The boxes at the left lower quadrant contain the semi-quantitative results of RT-PCR. (a, c, e and g) Expression of nestin protein in normal duodenum and (b, d, f and h) ampullary adenocarcinoma. (a and c) Weak expression of nestin in normal duodenal mucosa and (b and d) strong or mild expression of nestin in T2N0, stage IB ampullary adenocarcinoma of the same patient. (b) Strong expression of nestin in adjacent adenoma is shown to the left side of the arrows. (e) Weak expression in normal duodenal mucosa and (f) negative expression in T3N1, stage IIB ampullary adenocarcinoma. The nestin mRNA is downregulated in stage IIB cancer (T3N1) in RT-PCR (Fig. 1a) and (f) immunoreactivity of nestin is nearly undetectable in cancer with (e) preservation in normal duodenum. (g) Weak expression in normal duodenal mucosa and (h) weak expression in T4N1, stage III ampullary adenocarcinoma. The IHC results in T4N1 disease revealed equal density of nestin in cancer and normal duodenum, which was the same as the results in RT-PCR. Expression of nestin protein in IHC staining was correlated with detection of nestin mRNA in RT-PCR.

IHC staining of nestin was performed to confirm the results of the RT-PCR and indicated that intratumor lymphatic ducts or vessels also exhibited nestin expression (Fig. 1c). The immuno-reactivity of nestin was primarily detected in the cytoplasm of cancer cells; Fig. 1d displays the strongest immunoreactivity of nestin (IRS 12 points). Fig. 2 shows examples of IHC staining, which were matched with the RT-PCR results. The level of nestin expression in cancer cells was defined as negative (IRS 0–1 points; 48 patients), weak (IRS 2–3 points; 26 patients), mild (IRS 4–8 points; 25 patients), or strong (IRS 9–12 points; 3 patients). Patients who exhibited strong nestin expression were grouped with those with mild expression as these groups were small. Weak nestin expression was correlated with negative lymphovascular invasion, decreased perineural invasion, absence of pancreatic invasion, free resection margin and an early stage (Table I). Patients with mild or strong nestin expression had a small tumor, pancreatic invasion, a microscopically positive margin and advanced cancer stage (Table I).

### Correlation of nestin expression with the outcomes of the patients

Patients with weak nestin expression had a decreased incidence of recurrence, and particularly local recurrence or recurrence within the 12 months after operation (Table II). Patients with negative or mild to strong nestin expression experienced an increased risk of recurrence (Table II). Patients with weak nestin expression had a more favorable prognosis (5-year recurrence-free survival rate of 63.5%) than those with negative or mild to strong nestin expression (31.0 and 43.6%) (Fig. 1e). The regimens of adjuvant therapy were similar among the patients with negative, weak or mild to strong nestin expression (data not shown). A multivariate analysis was conducted to examine the survival predictors (Table III). Histological differentiation, tumor stage, nodal metastasis, pancreatic invasion, resection margin, and American Joint Committee on Cancer (AJCC) tumor, node and metastases (TNM) staging system have been used as predictors in previous studies (3–5). In our results, resection margin and nestin expression were prognostic predictors. A microscopically positive margin predicted recurrence; however, weak nestin expression predicted a favorable prognosis (P=0.030) (Table III).

### Gene interaction network of nestin in ampullary adenocarcinoma

**Figure 3:**
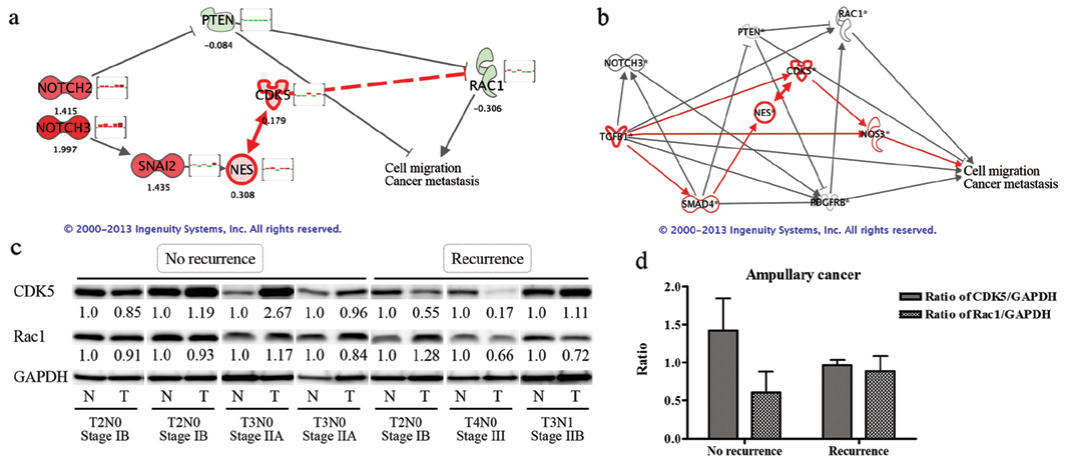
Upstream mediators and downstream signaling of nestin. (a and b) Gene network is generated through the use of Ingenuity Pathway Analysis (IPA). Gene network is represented as nodes and lines between two nodes. Node shapes symbolize the functional class of the gene product: inverted bell, cytokine and growth factor; hook, enzyme; trefoil, kinase; dumbbells, transcription regulator; tilted pot, phosphatase; circle, complex or other. The bar graphs to the right of the particular molecules depict the relative fold-change of the particular gene and the bar from left to right is represented as different patients. Red bar indicates that the gene is higher in cancer than normal and a green bar indicates that it is lower in cancer than normal. Bold nodes with red rims represent genes associated with nestin. (a) The expression pattern of nestin is opposite to the pattern of RAC1. (b) Activation of multiple oncogenic pathways and knockdown of tumor suppressors form a complex network to induce cell migration and cancer metastasis. (c) Detection of cyclin-dependent kinase 5 (CDK5) and Rac1 by western blotting. GAPDH served as a quantitative control. The fold-change of CDK5/GAPDH or Rac1/GAPDH is labeled below the band. N, normal tissue; T, tumor tissue. (d) Ratio of CDK5/GAPDH or Rac1/GAPDH expression was compared between patients with or without recurrence. An increased ratio of CDK5/GAPDH and a decreased ratio of Rac1/GAPDH were detected in patients without recurrence but not in patients with recurrence.

A microarray analysis of paired samples was conducted to identify the interacting genes associated with nestin. A total of 5 patients with ampullary adenocarcinoma were enrolled. One of the 2 patients with T2N0 (stage IB cancer) and 2 of the 3 patients with T3N0 (stage IIA cancer) developed recurrence. The Notch pathway is located upstream of nestin (22) and the NOTCH2 and NOTCH3 genes were overexpressed in ampullary cancer (Fig. 3a). The interaction between nestin and CDK5 modulates downstream molecules, including RAC1 and NOS3 [epithelial nitric oxide synthase (eNOS)] (23–25). Four of the 5 patients exhibited increased nestin expression and 3 of the 5 patients exhibited decreased CDK5 or RAC1 expression. The RAC1 pathway was suppressed among certain patients with early ampullary cancer.

In advanced cancer, multiple oncogenic pathways were activated and tumor suppressors were inhibited (Fig. 3b). The transforming growth factor-β1 (TGF-β1)/Smad pathway enhances cell migration through nestin/CDK5 signaling. Activation of the platelet-derived growth factor receptor (PDGFR) pathway and supression of phosphatase and tensin homolog (PTEN) signaling promote cell migration. Regarding the patients with negative nestin expression, the activation of NOTCH, TGF-β1 or PDGFR pathways could induce cancer metastasis (Fig. 3b).

Fresh tissue specimens of paired cancer and normal mucosa from 7 patients were examined by western blotting, including 4 patients without recurrence and 3 with recurrence (Fig. 3c). CDK5 and Rac1 were key proteins in the downstream signaling of nestin, and quantitative results of the CDK5/GAPDH or Rac1/GAPDH ratio are shown in Fig. 3d. An increased ratio of CDK5/GAPDH with a decreased ratio of Rac1/GAPDH was detected in patients without recurrence; however, the phenomenon disappeared in patients with recurrence. The result of the western blotting confirmed that the RAC1 pathway was suppressed in certain patients with early ampullary cancer.

## Discussion

Ampullary adenocarcinoma is the most common malignancy in the small intestine. This is the first study to discuss nestin expression in ampullary adenocarcinoma; the findings indicated that nestin expression was low in normal duodenum, upregulated in the early stages of cancer, downregulated in the intermediate stages of cancer, and upregulated in locally advanced ampullary adenocarcinoma. Patients with nestin expression in cancer cells had varied prognosis: those with weak nestin expression had a favorable recurrence-free survival, whereas those with mild to strong nestin expression had an unfavorable survival.

In normal physiological development, nestin is expressed in neurogenic or myogenic cells, and is replaced by tissue-specific intermediate filaments after differentiation (26). In pathological situations, nestin expression is reinduced (27). Nestin is overexpressed in cancer and is correlated with nodal metastasis and cancer invasiveness (18–20). Nestin was found to modulate metastasis in cancer and cell migration was inhibited by nestin-shRNA (28). Su *et al* (29) confirmed that nestin overexpression enhances cell migration and EMT through the TGF-β1/Smad4-mediated pathway. In this study, nestin mRNA was overexpressed in the early stages of cancer without lymph node metastasis and in the late stages of cancer with lymph node metastasis, but not in the intermediate stages of cancer (Fig. 1). The results were reconfirmed using IHC staining (Fig. 2). Weak nestin expression was correlated with favorable tumor features (Table I). The patients with weak nestin expression demonstrated a better recurrence-free survival rate (Fig. 1e and Table III). However, mild or strong nestin expression was correlated with unfavorable tumor characteristics and an increased risk of recurrence (Table II). This result contradicted previous studies that have explored nestin expression among other cancers (18–20). We speculate that nestin exhibits various functions in the early and advanced stages of ampullary adenocarcinoma. Certain oncogenic proteins involved in ampullary cancer exhibit functions distinct from those exhibited in other cancers. EpCAM is expressed in normal epithelium and is upregulated in epithelial cancer. EpCAM regulates c-myc and cyclins to enhance cell proliferation (30). However, the loss of EpCAM is related to an aggressive tumor phenotype of ampullary cancer, which is different from other types of cancer (10). The findings in the present study suggest that nestin plays various roles in early and in advanced ampullary adenocarcinoma.

Nestin is regulated by CDK5, and CDK5 regulates apoptosis, senescence, angiogenesis, differentiation and migration (31,32). Accumulation of CDK5 under oxidative stress triggers apoptosis and cell death (31). However, activation of CDK5 in cancer induces nestin reorganization, and inhibition of CDK5 reduces tumor growth and metastasis (23,33). The varying behaviors of CDK5 may explain why nestin plays various roles in early and in advanced ampullary adenocarcinoma.

A microarray analysis that compared cancerous and normal cells was conducted to explore the regulators of nestin (Fig. 3). A small GTPase protein, RAC1, is connected with nestin through CDK5 (Fig. 3a). RAC1 facilitates the proliferation of intestinal stem cells, formation of intestinal adenoma, initiation of colorectal cancer and migration of cancer cells (34,35). Oxidative stress suppresses the kinase activity of RAC1, induces activation of CDK5 and results in senescence of cells (24). Fig. 3a shows inconsistent patterns of nestin, CDK5 and RAC1 expression in the cDNA microarray and western blotting is shown in Fig. 3c. In the patients without recurrence, an increased CDK5/GAPDH ratio and a decreased Rac1/GAPDH ratio were detected (Fig. 3c and d). Lacking adequate neovascularization, hypoxia may develop during growth of a primary tumor in early ampullary cancer; thus, nestin/CDK5 temporarily suppresses RAC1. The cell migration and cancer metastasis are transiently suppressed and the tumor grows locally. This may be the reason that patients with weak nestin expression in ampullary adenocarcinoma had a favorable prognosis after undergoing radical resection.

The function of nestin/CDK5 changes in advanced cancer (33). Nestin behaves as a stemness protein to induce proliferation, migration and metastasis (28,29). In pancreatic cancer with nestin overexpression, a K-ras mutation was found to induce persistent activation of CDK5 (36). Suppression of the nestin/CDK5 complex reduces proliferation, migration and metastasis in cancer (33,36). In this study, nestin was a predictor of distant metastasis and poor prognosis in the ampullary cancer patients with mild to strong nestin expression (Table II and Fig. 1e). Inhibition of nestin in the select patients with ampullary adenocarcinoma could provide a therapeutic solution.

Furthermore, the activation of multiple oncogenic pathways and the knockdown of tumor suppressors form a complex network in cancer cells. The existence of crosstalk between the WNT, BMP, Hedgehog and Notch pathways in cancer has been verified. Accumulation of mutations in these pathways contributes to tumor heterogeneity (37). Furthermore, the inhibition of Notch attenuates nestin expression (22,38). In this study, the microarray data indicated overexpression of the NOTCH2 and NOTCH3 genes (Fig. 3a). Deregulation of the PDGFR mutation, activation of the TGF-β1/Smad pathway and inhibition of PTEN also enhance cell migration (Fig. 3b). Among patients with negative nestin expression, the activation of Notch, TGF-β1 or PDGFR pathways and suppression of PTEN may induce cancer metastasis.

Nestin expression was upregulated in the early and locally advanced stages of ampullary adenocarcinoma, but was not upregulated in the intermediate stage. Weak nestin expression was correlated with favorable tumor characteristics and a good prognosis. The nestin/CDK5 complex may suppress RAC1 and contribute to a favorable prognosis in patients with weak nestin expression. Activation of multiple oncogenic pathways in patients who exhibited or did not exhibit nestin overexpression increased the likelihood of a poor prognosis. Blocking nestin may prevent metastasis in patients with strong nestin expression.

## Figures and Tables

**Table I:** Correlation of nestin expression with the patient clinicopathological findings in ampullary adenocarcinoma.

		Nestin expression by IHC staining			
			
	All cases (n=102)	Negative (n=48)	Weak expression (n=26)	Mild or strong expression (n=28)	P-value
Age at surgery (years)^a^	65 (32–90)	62 (32–90)	64 (36–78)	67 (35–83)	0.131
Gender, n (%)					0.350
Female	41 (40)	19 (40)	8 (31)	14 (50)	
Male	61 (60)	29 (60)	18 (69)	14 (50)	
Tumor type, grossly, n (%)					0.419
Polypoid	59 (58)	25 (52)	14 (54)	20 (71)	
Ulcerative	21 (21)	10 (21)	6 (23)	5 (18)	
Mixed	22 (22)	13 (27)	6 (23)	3 (11)	
Histological differentiation, n (%)					0.568
Well	45 (46)	18 (38)	12 (50)	15 (56)	
Moderate	47 (48)	26 (54)	11 (46)	10 (37)	
Poor	7 (7)	4 (8)	1 (4)	2 (7)	
pT status, n (%)					0.051
pT1	12 (12)	3 (6)	4 (15)	5 (18)	
pT2	42 (41)	16 (33)	16 (62)	10 (36)	
pT3	32 (31)	18 (38)	5 (19)	9 (32)	
pT4	16 (16)	11 (23)	1 (4)	4 (14)	
Tumor size (cm)^a^	2.4 (0.7–8.0)	2.5 (0.8–8.0)	2.5 (1.0–6.5)	1.7 (0.7–4.0)	0.010
Pancreatic invasion, n (%)					0.018
Negative	54 (54)	21 (44)	20 (77)	13 (50)	
Positive	46 (46)	27 (56)	6 (23)	13 (50)	
Lymphovascular invasion, n (%)					0.003
Negative	40 (50)	11 (28)	14 (70)	15 (60)	
Positive	44 (50)	28 (72)	6 (30)	10 (40)	
pN status, n (%)					0.508
pN0	55 (60)	26 (55)	16 (70)	13 (62)	
pN1	36 (40)	21 (45)	7 (30)	8 (38)	
Perineural invasion, n (%)					0.030
Negative	46 (68)	15 (52)	14 (88)	17 (74)	
Positive	22 (32)	14 (48)	2 (12)	6 (26)	
Resection margin, n (%)					0.031
Free	91 (89)	41 (85)	26 (100)	24 (86)	
Microscopically positive	11 (11)	7 (15)	0	4 (14)	
AJCC TNM stage^b^, n (%)					0.038
Stage I	45 (44)	15 (31)	17 (65)	13 (46)	
Stage II	41 (40)	22 (46)	8 (31)	11 (39)	
Stage III	16 (16)	11 (23)	1 (4)	4 (14)	

a Values are expressed as median (range).

b AJCC TNM stage, American Joint Committee on Cancer tumor, node, metastases staging system.

IHC, immunohistochemistry.

**Table II:** Recurrence in patients with ampullary adenocarcinoma after radical resection, as compared with nestin expression.

	Nestin expression by IHC staining			
		
	Negative (n=48)	Weak expression (n=26)	Mild or strong expression (n=28)	P-value
Liver metastasis, n (%)	17 (35)	6 (23)	5 (18)	0.199
Local recurrence, n (%)	21 (44)	4 (15)	12 (43)	0.024
Peritoneal carcinomatosis, n (%)	9 (19)	2 (8)	2 (7)	0.219
Bone metastasis, n (%)	5 (10)	2 (8)	1 (4)	0.523
Other metastasis^a^, n (%)	11 (23)	2 (8)	5 (18)	0.217
Late recurrence, n (%) (> postoperative 12 months)	10 (21)	5 (19)	11 (39)	0.155
Early recurrence, n (%) (≤ postoperative 12 months)	23 (48)	5 (19)	7 (25)	0.020
Subtotal^b^, n (%)	33 (69)	10 (38)	18 (64)	0.035

a Including brain, lung and ovary metastases.

b Some patients developed more than one type of recurrence.

IHC, immunohistochemistry.

**Table III:** Multivariate analysis of the prognostic factors for recurrence-free survival in patients with ampullary adenocarcinoma who underwent radical resection.

	HR	95% CI	P-value
Age (years)	1.008	0.985–1.032	0.499
Gender			0.874
Female	1		
Male	0.958	0.561–1.634	
Resection margin			0.002
Free	1		
Microscopically positive	3.080	1.523–6.229	
Nestin expression in IHC			0.061
Negative	1		
Weak positive	0.446	0.215–0.926	0.030
Mild or strong positive	0.636	0.350–1.159	0.139

HR, hazard ratio; CI, confidence interval; IHC, immunohistochemistry.

## Abbreviations

AJCC TNM stage: American Joint Committee on Cancer tumor, node and metastasis staging system
CDK5: cyclin-dependent kinase 5
CSC: cancer stem cell
EMT: epithelial-mesenchymal transition
eNOS: endothelial nitric oxide synthase
IHC: immunohistochemistry
PDGFR: platelet-derived growth factor receptor
PTEN: phosphatase and tensin homolog
Rac1: Ras-related C3 botulinum toxin substrate 1
RT-PCR: reverse transcription-polymerase chain reaction
TGF-β: transforming growth factor β
